# C-Terminal Binding Protein: A Molecular Link between Metabolic Imbalance and Epigenetic Regulation in Breast Cancer

**DOI:** 10.1155/2013/647975

**Published:** 2013-05-20

**Authors:** Jung S. Byun, Kevin Gardner

**Affiliations:** Genetics Branch, Centers for Cancer Research, National Cancer Institute, 41 Library Drive, Bethesda, MD 20886, USA

## Abstract

The prevalence of obesity has given rise to significant global concerns as numerous population-based studies demonstrate an incontrovertible association between obesity and breast cancer. Mechanisms proposed to account for this linkage include exaggerated levels of carbohydrate substrates, elevated levels of circulating mitogenic hormones, and inflammatory cytokines that impinge on epithelial programming in many tissues. Moreover, recently many scientists have rediscovered the observation, first described by Otto Warburg nearly a century ago, that most cancer cells undergo a dramatic metabolic shift in energy utilization and expenditure that fuels and supports the cellular expansion associated with malignant proliferation. This shift in substrate oxidation comes at the cost of sharp changes in the levels of the high energy intermediate, nicotinamide adenine dinucleotide (NADH). In this review, we discuss a novel example of how shifts in the concentration and flux of substrates metabolized and generated during carbohydrate metabolism represent components of a signaling network that can influence epigenetic regulatory events in the nucleus. We refer to this regulatory process as “metabolic transduction” and describe how the C-terminal binding protein (CtBP) family of NADH-dependent nuclear regulators represents a primary example of how cellular metabolic status can influence epigenetic control of cellular function and fate.

## 1. Introduction

The first written description of breast cancer was recorded in 3000 B.C. as an inscription in the Smith Papyrus that pictured ulcerating lesions of the breast, a condition for which there was no cure [1]. Though, as early as Hippocrates (460–375 BC), the general belief was that cancer initiated from natural causes, a fuller understanding of cancer did not emerge until the late nineteenth century where the development of higher resolution microscopy made the visualization of cells and tissue possible [2]. This event marked the birth of modern pathology and revealed that there are striking differences in the appearance of cancer cells when compared to the surrounding normal tissue. This difference or “otherness” of malignancy made it clear that cancer develops from a change or transformation of normal tissue, a difference that modern molecular biology reveals to be rooted in genomic “changes” or mutation to cellular DNA sequence [3]. It is now widely recognized that mammalian cells are constantly exposed to genotoxic stress from both endogenous and exogenous sources that threaten to change or mutate the human genome and thereby increase the risk of cancer. To address this threat, mammalian cells and tissues have evolved numerous mechanisms and pathways to identify and repair damage to the human genome [4]. These DNA repair pathways and the manner in which cells and tissues provide surveillance to identify and remove cells that have irretrievably lost their genomic fidelity constitute what is known as the DNA damage response (DDR) [3, 4], which is now recognized as a major criteria through which clinicopathological assessment and classification of breast cancer are defined [5–8]. 

## 2. The Molecular Stratification of Breast Cancer

A significant development in the clinicopathological assessment of breast cancer has been the recognition that breast cancer is a heterogeneous disease that can be stratified into relatively distinct entities or subtypes based on specific molecular parameters [6, 9, 10]. At least 5 different classes or “subtypes” are described, including luminal A, luminal B, human epidermal growth factor 2 (HER2) positive, basal-like, and claudin low [10]. Each of these subtypes has been shown to resemble or reflect distinct stages of mammary differentiation where the claudin low and basal-like represent the more primitive, receptor deficient, “triple negative” (lacking estrogen, progesterone, and HER2 receptors) spectrum of differentiation [11]. Most notably, a similar functional stratification exists with respect to DNA repair, where the less differentiated subtypes exhibit the greatest deficiencies in genome stability [5]. Since tumors with higher levels of genome instability typically show more aggressive behavior, it is clear that this stratification of breast cancer phenotypes and its functional correlation with DNA repair capacity have significant prognostic importance that will guide therapeutic strategies [6, 7, 12, 13]. These relationships are well demonstrated by the observation that the gene products of the early onset breast cancer genes BRCA1 [13, 14] and BRCA2 [15], whose germline loss or mutation confers a near 80% risk of developing breast cancer, are themselves DNA repair proteins [16]. Moreover, patients with germline depletion of BRCA1 give rise to tumors of the more primitive, triple negative, basal-like, and claudin-low subtypes [17, 18]. Similarly, sporadic tumors that show deficiencies in BRCA1 tend to be of the basal-like or claudin-low subtype, and estimates indicate that as many as 40% of nonhereditary or sporadic breast cancers show decreased expression of BRCA1 [19].

## 3. Obesity, Diabetes, and the Risk of Breast Cancer

It is estimated that obesity in United States accounts for nearly 15%–20% of cancer deaths [20, 21]. Cancer death rates from women who are obese are from 50% to 60% higher than women who are of normal weight. Breast cancer rates in postmenopausal women increase 30%–50% with obesity and are associated with more aggressive disease. In fact, very obese women with a body mass index (BMI) greater than 40.0 kg/m^2^ have a three times higher risk of death from disease compared to their much leaner (BMI < 20.5 kg/m^2^) counterparts [22]. Finally, as might be predicted, patients who are both obese and have germline BRCA1/2 mutations show significantly increased risk over either condition alone [23].

The relationship between obesity and breast cancer is complex. Most studies have focused on the abnormally elevated levels of circulating mitogenic hormones and inflammatory cytokines [22, 24, 25]. The rise in adipocyte size due to calorie excess causes increased release of free fatty acids and enhanced secretion of peptide hormones such as leptin, resistin, and tumor necrosis factor alpha (TNF*α*) and reduced release of adiponectin. Adipose cells also express significant levels of steroid hormone metabolizing enzymes. The high levels of TNF*α* and reduced level of adiponectin give rise to insulin resistance and type II diabetes [24, 25]. The elevated insulin levels result in increased production of insulin-like growth factor one (IGF-1), both of which promote cellular proliferation. Increased levels of steroid hormone metabolizing enzymes like aromatase in adipose cells convert circulating androgen precursor to estrogens. This rise in free circulating estrogen is further exacerbated by the reduced synthesis of the sex-hormone binding globulin (SHBG) due to the obesity-associated hyperinsulinemia [24–26]. 

## 4. The Molecular Cost of Metabolic Imbalance

To define the link between obesity and cancer we must begin by understanding the molecular impact or “cost” of metabolic imbalance. Conditions of excess calories or “overnutrition” have profound effects on cellular metabolism [26]. Oxidation of free fatty acids, glucose, and other carbon intermediates by beta-oxidation, glycolysis, and the tricarboxylic acid cycle transfers electrons primarily to nicotinamide adenine dinucleotide (NAD+) to produce NADH. The NADH produced in this fashion normally has several different fates including its oxidation in the presence of molecular oxygen via the mitochondrial electron transport chain to produce H_2_O and ATP. One well-understood consequence of the elevated NADH levels due to nutrient excess is the increased generation of reactive oxygen species (ROS) like superoxide (O_2_^−^) as a consequence of incomplete mitochondrial electron transfer during respiration [27, 28]. The ROS, thus generated, contributes significantly to the risk of malignant transformation by causing DNA damage.

## 5. NAD+/NADH and Cancer Metabolism

The NAD+/NADH redox imbalance of nutrient excess and obesity is reminiscent of the metabolic imbalance associated with the malignant proliferation of cancer, first described by Otto Warburg in 1927 [26, 103–111]. In his work, Warburg noted that cancer cells demonstrated high levels of glucose consumption and lactic acid production even though there was significant oxygen to sustain the respiratory production of energy by the oxidation of NADH via oxidative phosphorylation. The net result is an elevation in the steady state levels of cellular NADH. This shift in energetic carbon flux is referred to as the *Warburg Effect* and has been the focus of extensive investigation as a potential vulnerability of cancer metabolism (“cancer's sweet tooth”) that may be exploited for chemotherapeutic benefit [106, 107, 109, 111]. 

In addition to threats to genome integrity from elevated ROS, the shifts in the redox status and availability of NAD+, due to either calorie excess or the *Warburg Effect*, have profound influence on certain specialized classes of mammalian proteins that directly utilize oxidized or reduced NAD+ as cofactors, ligands, or substrates. Some of these factors have widespread impact on genome integrity by controlling the DNA damage response and include the Sirtuin family of Class III histone deacetylases [112, 113], the PARP family of poly ADP ribosyl-transferases [114–116], and the C-terminal binding protein (CtBP) class of transcriptional repressors [117–120]. The relative *K*_*M*_ of the Sirtuin and PARP family are in the 50–200 micromolar range [121]. Since the levels of free cytoplasmic NAD+ are in the range of 500–800 micromolar, both the Sirtuin and PARP families are functioning at saturation. In contrast, the free cytoplasmic concentration of NADH is 1 micromolar, while the CtBP binding protein (CtBP) class of transcriptional repressors has binding affinity in the 100 nanomolar range [122]. Therefore, CtBP is likely to function as a true sensor of cellular metabolic status by sensing acute and chronic changes in cytoplasmic and nuclear levels of NADH. 

This review will summarize the role of CtBP in the maintenance of genomic homeostasis and describe how its activity links cellular metabolic status with genome stability and epithelial reprogramming in breast cancer and how this linkage has broader implications for other epithelial cancers.

## 6. C-Terminal Binding Protein Structure and Function

CtBP is expressed from two genes, *CtBP1* and *CtBP2*. Both of these genes give rise to many different isoforms, some displaying distinct functions. There are essentially four major CtBP isoforms: *CtBP1-L*, a shorter isoform of *CtBP1* (CtBP1-S) referred to as *CtBP3/BARS*, CtBP2 (including a *CtBP2-L* and shorter splice isoform *CtBP2-S*), and *RIBEYE*, a variant that contains a large N-terminal domain and is transcribed from an alternate *CtBP2* promoter [119]. CtBP was first identified as a phosphoprotein that interacted with the C-terminal protein sequences encoded in exon 2 of the oncogenic adenovirus 2/5 E1A protein [123]. The CtBP protein was later found to function as a transcriptional corepressor when targeted to gene promoters through the C-terminal sequences of E1A [124]. This binding was mediated by a highly conserved 5-6 amino acids binding motif (PXDLSK) that adopted the conformation of a series of beta turns in solution [125]. The PXDLS binding motif was later found in a variety of transcriptional repressors across different species, including drosophila transcriptional regulators that play broad roles in tissue morphogenesis, like zfh-1, hairy, knirps, giant, kruppel and snail and its mammalian homologues snail, and ZEB1/2 [29–32, 126]. A search for other proteins that interact with CtBP led to the identification of C-terminal binding protein interacting protein (CtIP) [127], a protein that was later implicated in having a significant role in maintaining genome stability through its interaction with the early onset breast cancer gene, *BRCA1* [35], indicating that CtBP could bind to a diverse array of factors with distinct and overlapping molecular functions. This link was later expanded when it was found that Rb pocket binding LXCXE motif within CtIP enabled it to form higher order complexes with CtBP and Rb family members via a separate domain containing the common PXDLS motif indicating that CtIP/CtBP complexes represent a corepressor assembly that could recruit tumor suppressors like Rb in the context of histone deacetylase activity. Subsequently, CtBP began to show up in a variety of other protein interaction screens as binding partners for several different transcriptional regulators that control diverse cellular programs including Net (*ELK4*), a member of the ternary complex family involved in regulation of* Fos* and other immediate early gene expressions through the serum response element, and the transcriptional repressor KLF8 (*ZNF741*) [36, 37].

## 7. Early Clues of Role for CtBP in Development

CtBP is well conserved, and forms of CtBP are expressed from men to flies, worms, and plants [118, 128, 129]. Consistent with its role in tissue morphogenesis, first demonstrated in drosophila, several studies have identified a role for CtBP in driving mammalian epithelial programming where the cellular adhesion molecule E-cadherin is a major target of repression through the recruitment of CtBP/ZEB complexes to multiple E-box transcription factor binding sites (TFBS) in the E-cadherin (*CHD1*) promoter [130]. This study also shows that CtBP inhibits cell anoikis, suggesting a prominent role for CtBP in promoting the early stages of epithelial-to-mesenchymal transition [130, 131]. Other lines of evidence are beginning to suggest significant roles for CtBP in epithelial reprogramming. For instance, gene expression studies in CtBP depleted cells reveal that multiple epithelial and proapoptotic gene pathways are regulated by CtBP [38]. Subsequently, several other genes have been found to be transcriptionally regulated by CtBP, including the telomerase protein and RNA components, *TERT* and *hTERC* [132], the notch target gene *Hey1* [74], and the brain-derived neurotrophic factor (*BDNF*) promoter through binding the REST transcription factor [133]. Transforming growth factor beta (TGF-*β*) plays a ubiquitous and multipotent role in regulating tissue morphogenesis. Under certain conditions, its influence is antiproliferative; yet under others, it can promote tumor progression and invasion [134]. The CtBP interaction with the transcriptional regulator Evi-1 protein, known for inducing blocks to differentiation that promote leukemogenesis, is also known to inhibit signaling through TGF-*β* by associating with Smad3 containing complexes and recruiting CtBP [39]. This interference with TGF-*β* signaling is also facilitated through the interaction of CtBP with inhibitory Smad 6 [60]. Accordingly, Evi-1 induced transformation of Rat1a fibroblast requires CtBP, the first indication that CtBP plays a direct role in cellular transformation [135]. Similarly, overexpressed and t(3;21) chimeric fusions of Evi-1 also function through CtBP to block differentiation and promote leukemogenesis [136, 137]. CtBP transgenic studies show that CtBP plays roles in a vast variety of developmental functions [138]. While CtBP2 deletions are an embryonic lethal (E10.5), mice with CtBP1 disruption are viable and fertile but die early [118, 138].

## 8. CtBP and NADH

It was not until nearly a decade after its first discovery that CtBP was found to be an NADH regulated dehydrogenase of the well-conserved D2 hydroxyacid dehydrogenase class that undergoes a conformational change in association with either NAD+ or NADH [139]. Moreover, it was found that residues associated with the active site are linked to the ability of the dimeric components to bind to the PXDLS peptide motif on its binding partners [139]. Like other D2 hydroxyacid dehydrogenases (e.g., GAPDH), CtBP was found to form higher order oligomers and increase its interaction with PXDLS containing protein domains in the presence of NADH and NAD+ [140]. Moreover, its interaction with PXDLS containing peptides slowed the catalytic activity [140]. Notably, the binding affinity of CtBP was over 100-fold higher for NADH than NAD+ suggesting a substantial role for CtBP as a metabolic sensor of redox status [122, 141]. However, ablation of enzymatic activity by mutations at histidine-315 showed no effect on CtBP transcriptional regulation, suggesting that NADH/NAD+ binding, not the dehydrogenase activity of CtBP, was necessary for its repressor activity [38, 142]. Nonetheless, a developmental study in drosophila revealed distinct phenotypes for CtBP with impaired enzymatic activity; so, the identity and role of the true substrate for the CtBP dehydrogenase remain a mystery [143].

## 9. CtBP and Regulation of the Epigenome

Several reports demonstrate that CtBP forms complexes with a variety of epigenetic regulators or corepressor complexes that recruit epigenetic regulators. Initial studies show that CtBP interacts with consensus binding motifs on class II histone deacetylases (HDAC 4, 5, and 7) and the corepressor protein MEF2-interacting transcription repressor (MITR/HDAC9), a structural scaffold that associates with other HDACs, through amino terminal sequences [40]. The class I histone deacetylases (HDACs, 1, 2, and 3) are also found to associate with CtBP through various multicomponent complexes [42, 144]. In multiple studies, these complexes were found also to contain several different types of epigenetic regulators, including histone methyltransferases G9a and EHMT1; the G9a and EHMT1 binding zinc finger protein, WIZ, that bridges interaction with CtBP; the histone demethylase LSD1; an actin-related component of the SWI/SNF complex, ArpN*α*; CoRest (RCOR1), a corepressor protein that interacts with REST transcription factor; CDYL, a component of the polycomb regulatory complex 2 (PRC2) that bridges interaction between repressive Histone H3K27Me3 modifications and the EZH2 histone H3K27 methyltransferase; and a component of the polycomb regulatory complex I (PRC1), CBX4 [42–44, 46]. Since both G9a and CBX4 interact with DNA methyl-transferases [145–148], it is likely that some CtBP complexes will also contain DNA methyl-transferases. Recent findings indicate that the histone acetyl-transferase, p300, also forms complexes with CtBP. In the CtBP:p300 complex, CtBP interaction with the p300 bromodomain represses p300 HAT activity [45]. The BCL6 corepressor BCOR-L1 associates with CtBP in combination with class II HDACs (HDAC 4, 5, and 7) to repress target genes like E-cadherin [47]. The estrogen receptor corepressor, Rip140, forms a complex with CtBP to participate in control of hormone regulated genes [48]. Transcriptional repression through GATA2 and GATA3 is mediated by combined association of the corepressor, friend of GATA (FOG) with CtBP to block adipogenesis [49]. Another pathway through which CtBP controls adipocyte growth and differentiation is through the transcription factor PRDM16 that recruits CtBP to shut down genes that promote white adipose cell growth and differentiation and eventually exchanges CtBP factors for PGC-1*α* and PPAR*γ* to drive the expression of brown fat genes [50]. Finally, it has been shown that the p53 gene product and regulator hmd2 can act as a corepressor at p53 regulated genes to recruit CtBP to mediate transcriptional repression [51].

Other DNA binding transcription factors regulated by CtBP include hypermethylated in cancer (HIC1), which forms a complex with CtBP that regulates SIRT expression; ZNF36, which is involved in the regulation of estrogen controlled genes; BCL6, which binds directly to CtBP to autoregulate its own transcription; the corepressor BCL3 whose association with DNA bound NF-kappa B dimers requires CtBP for transcriptional repression; Ikaros, a transcription factor necessary for lymphoid development, whose repressive activity requires a direct interaction with CtBP through an N-terminal PXDLS motifs and an interaction between CtBP and Sin3A; the TCF-4 of Wnt signaling whose physiological repression of CtBP target genes is lost in cancers that are deficient in mismatch repair and express TCF-4 isoforms incapable of binding CtBP [53–56, 58, 149–151].

## 10. Posttranslational Regulation of CtBP

CtBP protein undergoes dynamic posttranslational regulation (see Table 2). The changes influence either the stability or the subcellular localization of CtBP. CtBP2 and CtBP1 readily hetero- and homodimerize; however, only CtBP2 has a nuclear localization signal that allows it to translocate to the nucleus. Thus, CtBP1 must either enter the nucleus as a heterodimer with CtBP2 or through the formation of a complex with BKLF (KLF3) or other factors [57]. This mode of translocation is heavily dependent on CtBP1 dimerization so decreases in dimerization; through decreased availability of NADH can result in retention of CtBP1 in the cytoplasm [57]. The nuclear splicing factor Pnn/Drs has recently been found to be recruited to gene promoters by CtBP to influence splicing [152]. This interacting Pnn/CtBP complex also plays a role in sequestering CtBP in nuclear speckles to relieve transcriptional repression of CtBP-targeted genes [59]. Finally, the subcellular localization of CtBP is dynamically controlled by posttranslational modifications. Sumoylation of CtBP1 at lysine K428 results in increased nuclear retention [98]. Similarly, acetylation of CtBP2 by p300/CBP on lysine residues K6, K8, and especially K10 results in nuclear retention [153]. Interestingly, sumoylation of CtBP1 was inhibited in the cytoplasm by the PDZ protein nNOS resulting in greater cytoplasmic retention [78, 98]. The total levels of CtBP1 sumoylation appear low, and so the relative contribution of this modification to CtBP regulation remains unclear; however, CBX4 (see earlier), PIAS1, PIASx*α*, and PIASx*β* have been shown to be the likely E3 ligases involved [154–156]. The role of CBX4 in the regulation of CtBP1 remains complicated as CBX4 promotes complex formation between CtBP1 and ATK1, where ATK1 dependent phosphorylation of CtBP1 results in decreased dimerization and increased ubiquitination with subsequent proteasomal degradation [96]. Interestingly, CtBP1 is also phosphorylated on Ser-158 by AMP Kinase (AMPK) which results in decreased repressive function, suggesting a novel mechanism through which CtBP activities are controlled by nutrient stress [93].

Two previous reports have shown that the *adenomatous polyposis coli* gene (APC) interacts with CtBP and regulates its degradation through a proteasomal pathway [61–63, 85]. In fact, degradation or loss of CtBP was proposed as a necessary step in the evolution of colonic adenomas in a zebrafish model [63]. Another tumor suppressor gene that is associated with CtBP degradation is alternative read frame tumor suppressor gene ARF whose association with CtBP leads to its degradation [157]. Phosphorylation of CtBP on Ser-422 by homeodomain interaction protein kinase 2 (HIPK2) leads to its ubiquitination and proteasomal degradation in response to UV irradiation [65]. Similarly, c-jun NH2 terminal kinase 1 (JNK1) also phosphorylates CtBP on Ser-422 [66] suggesting that the induction of stress pathways may be a common mechanism to reduce the level of CtBP posttranslationally. Phosphorylation of CtBP on serine 158 by the p21 activated kinase PAK1 triggers relocalization to the cytoplasm [97]. Recent studies indicate that phosphorylation of CtBP on T144 by cyclic-AMP dependent kinase (PKA) leads to increased dimerization [92]. The interaction of CtBP with X-linked inhibitor of Apoptosis (XIAP) also leads to its ubiquitination and degradation. Though the mechanism of degradation has yet to be fully described, the removal of phosphorylated CtBP from promoter bound locations is thought to require the action of the transducin beta family of chaperone-like molecules, TBL1 [67]. Finally, very recently the C-terminus of Hsc70-interacting protein was found to interact with CtBP2 leading to its ubiquitination and subsequent degradation in the proteasome [95].

## 11. Moonlighting Functions of CtBP in the Cytoplasm

The fission and fusion of biological membranes during intracellular trafficking of membrane bound vesicles and structures support broad programs of endocytosis and exocytosis as essential cellular functions. Both forms of CtBP1 (CtBP1-L and the CtBP3/BARS/CtBP1-S) have cytoplasmic functions that remain to be clearly defined but appear to be linked to Golgi membrane fission and homeostasis [119, 158]. CtBP3/BARS is reported to have an essential role in regulating membrane fission in the Golgi tubular network and has also been implicated in mitotic partitioning of the Golgi apparatus [159, 160]. Though the mechanism and putative enzymatic activity exerted by CtBP in this processes remain unresolved [161, 162], there is general agreement for a central role of CtBP in the formation of vesicular and tubular membrane carriers that ferry membrane bound components to different intracellular compartments [163]. How this function may impact on the nuclear function of CtBP and how such activity influences the role of CtBP in development and oncogenesis remain unclear. Some reports suggest that loss of CtBP3/BARS is associated with decreased surface expression of the FAS/CD95 [164]; therefore, this mechanism may have a role in cellular survival strategies. However, given the known role of CtBP in influencing cellular reprograming and antagonizing the epithelial phenotype [118], it is tempting to speculate that this property of CtBP could have a role in defining epithelial polarity [165–167]. The partitioning of the Golgi during mitosis may also have a significant role in promoting formation and orientation of the mitotic spindle and thus could influence the asymmetric division necessary for the formation of stratified epithelia and the maintenance of pluripotent stem cell pools [168, 169]. Similar membrane trafficking promoted by CtBP3/BARS may have a role in maintaining the basolateral and apical polarity of epithelial cells during tissue morphogenesis, regeneration, and wound healing. All of these events are disrupted and deregulated in cancer. Finally, it is tempting to speculate that secretory tissues that must undergo cyclic proliferation and involution or repair (e.g., breast, endometrium, and colon) may be particularly dependent on both the nuclear and cytoplasmic functions of CtBP.

## 12. CtBP and Oncogenesis

Since its discovery in 1993, many studies provide evidence that CtBP plays an expanded role in the evolution and progression of cancer controlling gene expression through a variety of transcriptional regulators and gene networks (Table 1). Many of these networks are associated with malignant behavior in a variety of cell types. CtBP recruitment and transcriptional targeting of multiple genes important in hematopoietic differentiation, including *Evi-1*, *BCL3*, *BCL6*, *GATA1*, *GATA2/3*, FOG1, and Ikaros strongly implicate prominent roles for CtBP in the incidence and progression of erythroid, lymphoid, and myeloid malignancies [39, 49, 54, 56, 58, 136, 170, 171]. Similarly, the interaction of CtBP with a variety of developmentally regulated genes that control various processes in tissue development, like EMT, strongly implicates a significant role for CtBP in the incidence, growth, and progression of epithelial cancers [38, 130, 172]. As mentioned previously, the control of CtBP protein levels by APC was one of the earliest indications of a link between CtBP and epithelial cancers [62, 85, 173]. In fact, the earlier studies that linked CtBP function to EMT were carried out in lung and colonic cell lines and tissues [174, 175]. These have been augmented by studies with patient tissue showing correlation between CtBP expression and malignancy. Nadauld et al. showed that CtBP could be linked to impaired differentiation in colon cancer because it decreased the production of retinoic acid through repression of retinol dehydrogenase [62]. In this study, the investigators were able to show that, in colonic biopsies from patients with familial adenomatous polyposis (FAP) (germline deficiencies in APC, which induces CtBP degradation), adenomas showed elevated levels of CtBP1 that was correlated with reduced levels of retinol dehydrogenase expression [62]. Deng et al. found that CtBP1 expression was elevated in a large percentage patient melanomas [176]. Increased expression of both CtBP1 and CtBP2 has been seen in tumors from patients with head and neck squamous cell cancers [177, 178]. A very recent study reported an elevation of CtBP2 in ovarian cancer and suggested that CtBP2 expression could be used as a marker for patients that are more likely to respond to epigenetic therapy utilizing histone deacetylase inhibitors [179]. Another group at the University of Michigan recently reported that CtBP1 was overexpressed and mislocated in metastatic prostate cancer and suggested a prominent role for CtBP1 in the progression of prostate cancer [180]. Several recent studies provide strong evidence indicating that elevated CtBP expression and activity may play a significant role in human breast cancer [172, 181–185]. In each study, increased CtBP expression was found in malignant as opposed to nontransformed patient samples. Yet, a systematic profiling of the network of genes controlled by CtBP in human breast cancer and the implication of that control in breast cancer evolution and outcome has been lacking.

## 13. Genome-Wide Profiling of CtBP Interactions across the Breast Cancer Genome

**Figure 1:**
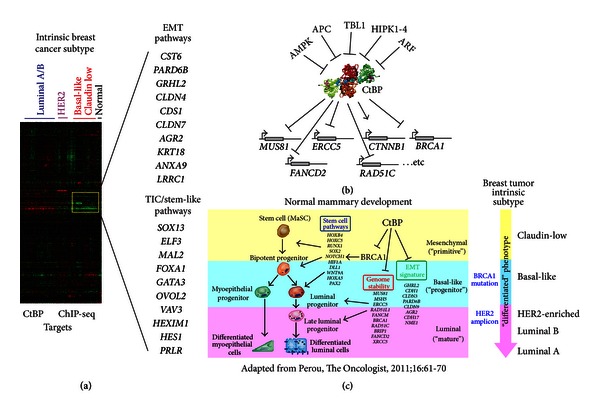
CtBP targets a network of interactions that control cellular reprogramming. (a) ChIP-Seq signature identifies multiple genes that are downregulated in breast cancer subtypes with primitive and mesenchymal features including the basal-like and claudin-low subtypes. (b) The CtBP targeted genes represent a network that exerts transcriptional control at the level of gene promoters and posttranslational stability of CtBP. (c) Representative genes targeted by CtBP influence cellular programming that correlate with primitive and more clinically aggressive intrinsic subtypes of breast cancer.

Beginning with the observation that CtBP could act as a metabolic sensor to control genome stability in breast cancer through the early onset gene *BRCA1*, one group at the National Cancer Institute has provided one of the first comprehensive genomic analyses of the interaction of CtBP with the human genome [185]. Using a combination of chromatin immunoprecipitation and deep sequencing (ChIP-seq), this group identified more than 1800 gene promoters that were potential candidates for transcriptional regulation by CtBP [185]. Remarkably, many of the genes that were targets of CtBP repression fell into 3 major classes: those genes that influenced genome stability (e.g., *BRCA1, BRIP1, RAD51C, ERCC5, PALB2, FANCD2, XRCC5*, and *FANCM*); those genes that controlled epithelial differentiation and are therefore down regulated during EMT (e.g., *CDH1, CST6, CLDN3, CLDN7, CLDN9, GRHL2, KRT18*, and *PARD6B*); those genes that are routinely repressed to maintain stem-cell like self-renewal and pluripotency (e.g., *HES1, OVOL2, FOXA1, GATA3, DKK1, CEBPb, RARG,* and *OAZ3*) [185] (see Figure 1). This was an important finding since these pathways represent significant hallmarks of cancer that play major roles in more aggressive forms of cancer through the promotion of uncontrolled growth, resistance to chemotherapy, and invasion and metastasis [186, 187]. As mentioned earlier, breast cancer subtypes stratify by morphology, molecular attributes, and prognosis along a hierarchy that reflects normal mammary epithelial development [188–190]. Tumors with high levels of phenotypic plasticity characterized by primitive, embryonic, or dedifferentiated mesenchymal features are usually of the basal-like and claudin-low subtypes. These tumors are typically estrogen receptor negative and the majority are also negative for the progesterone receptor and the human EGF receptor 2 (HER2) [189–191]. Such receptor negative tumors are often referred to as triple negative breast cancer (TNBC). These tumors also have the worst clinical outcomes with high mortality within the first five years after diagnosis [189–192]. Key molecular attributes of these tumors are increased genome instability, early invasion and metastasis, resistance to chemotherapy, and high expression of stem cell-like self-renewal pathways [189–194]. Strikingly, the CtBP target genes identified in this study could be readily used as a “signature” to predict poor clinical outcome based on metastasis free interval [185]. Moreover, using human breast cancer cell lines, the authors showed that disruption of CtBP, by either gene depletion or calorie restriction to lower endogenous NADH levels, increased DNA repair and diminished both the stem cell-like and invasive attributes of the cancer cells thus establishing a clear mechanistic link between CtBP, cellular metabolic status, and aggressive features of breast cancer. Finally, a screen of clinical samples from breast cancer patients revealed that those patients that had the highest expression of CtBP in their tumors had substantially shortened median breast cancer survival [185].

## 14. Conclusion

In this short review, we have described how, NADH, a central product of carbohydrate metabolism can act as a secondary messenger to control the activity of multiple different epigenetic regulatory complexes in human cells and how these modes of regulation, when disrupted by metabolic imbalance, can increase the risk of cancer of the breast and other types. The linkage in metabolism and epigenetic modification provides a novel window through which one could assemble newer strategies for therapy with particular focus on the nexus between metabolism and epigenetic modifiers. In this regard, it is reasonable to conjecture that certain metabolic therapies could have the potential to show efficacy in combination with epigenetic modalities in the treatment of breast and other cancers. The heightened excitement raised about the reported efficacy of the antidiabetic drug, metformin, as both a chemopreventive and treatment strategy in breast cancer is just one example [195, 196]. Other examples include the use of metabolic transducers like CtBP as a biomarker for efficacy in epigenetic therapy [179]. Since a direct effect of metformin is to increase AMPK activity, the finding that AMPK inhibits CtBP activity [93] certainly lends credence to such notion. Thus, new strategies to search for potential small molecules that may disrupt CtBP function could represent a novel form of epigenetic therapy where the target is gene-specific recruitment of chromatin modifying enzymes rather than the wholesale nonspecific repression of a whole enzyme class.

 While we specifically focus on CtBP, a pleiotropic regulatory complex controlling numerous epigenetic modifications, whose activities are “metabolically transduced” by NADH, there are clearly many other metabolic intermediates that influence epigenetic modifications and therefore provide a means through which aspects of metabolic status can be transduced to affect changes in gene expression through epigenetic regulation. Among them are the wide array of histone acetyl-transferases that utilize acetyl-CoA for producing epigenetic marks, the PARP family of proteins that modify chromatin by ADP-ribosylation, the Sirtuin family that consumes NAD+ during histone deacetylation, the Jumonji C family of histone methyl-transferases that utilize alpha-ketoglutarate, and the TET1/2 family of 5 mC oxidases that also consume alpha-ketoglutarate to influence DNA methylation. The manner in which the activities of these epigenetic regulators are coordinated with each other to sculpt and shape the epigenome in response to cellular metabolism represents a new area of investigation that will have broad implication not only in cancer but in a wide variety of human diseases.

## Figures and Tables

**Table 1:** CtBP interacting protein complexes.

Factor	Function	Ref
Zfh-1	Transcription repression	[29]
Hairy	Transcription repression	[30]
Knirps	Transcription repression	[31]
Giant	Transcription repression	[32]
Kruppel	Transcription repression	[32]
Snail	Transcription repression	[32]
ZEB1/2	Transcription repression	[33]
CtIP	Genome stability	[34]
BRCA1	Transcription repression	[35]
NET (ELK4)	Transcription repression	[36]
KLF8 (ZNF741)	Transcription repression	[37, 38]
Evi-1	Inducing blocks to differentiation	[39]
HDAC 4, 5, 7	Histone deacetylation	[40]
HDAC 1, 2, 3	Histone deacetylation	[41, 42]
G9a	Histone methyltransferase	[42]
EHMT1	Histone methyltransferase	[43]
WIZ	Transcription repression	[44]
Lsd1	Histone demethylase	[42]
ArpN*α*	Transcription repression	[45]
CoRest (RCOR1)	Transcription repression	[42]
CDYL	Transcription repression	[46]
CBX4	Transcription repression	[46]
P300	HAT inhibition	[47]
BCOR-L1	Transcription repression	[48]
RIP140	Hormone regulation	[49]
FOG	Transcription repression	[50]
PRDM16	Transcription repression	[51]
Hmd2	Transcription repression	[52]
HIC1	Regulate SIRT expression	[53, 54]
ZNF366	Estrogen control gene regulation	[55]
BCL3	Transcription repression	[56]
BCL6	Autoregulation of transcription	[56]
Ikaros	Transcription repression	[57]
Sin3A	Transcription repression	[57]
TCF4	Transcription repression	[58]
BKLF (KLF3)	Stability of subcellular localization	[59]
Pnn	Transcription Repression	[60]
APC	Degradation	[61–64]
ARF	Degradation	[65]
HIPK2	Ubiquitination, proteasomal degradation	[66]
JNK1	Phosphorylation	[67]
TBL1	Dephosphorylation	[67]
CDK7/CCNH	Post-translational stability	[68]
Huntingtin	Unknown	[69]
Glis2	Transcriptional repression	[70]
PLD1	Activation of macropinocytosis	[71]
Smad6	Transcriptional repression	[60]
Ataxin	CtBP antagonism	
PARP1	Corepressor complex	[72]
Sox6	Transcriptional repression	[73]
Spen	Transcriptional repression	[74]
BCoRL1	BCL6 transcriptional	[47]
	Co-repression	
Eos (IKaros family member)	Transcriptional repression	[75]
Acetylcholinesterase-S (AChE-S)	Antagonize CtBP transcriptional repression	[76]
SatB1	Co-repressor complex	[77]
nNos	Cytoplasmic localization	[78]
Tel/EVT6	Control of endothelial sprouting	[79]
ER-beta	Suppression of inflammatory response in CNS microglia and astrocytes	[80]
KLF12	Transcriptional repression	[81]
MLL	Transcriptional repression	[82]
HDGF	Transcriptional repression	[83]
KCNIP3/KCHIP	Calcium-dependent Transcriptional repression	[84]
MITR	Transcriptional repression	[40]

**Table 2:** CtBP regulators

Protein	Mode of regulation	Ref.
APC	Protein degradation	[61–63, 85]
	Proteasome dependent	
HIPK2	Phosphorylation-dependent	[86, 87]
	Protein degradation	
TBL1	Protein degradation	[88]
	Proteasome-dependent	
Ink4a/Arf	Protein degradation	[89, 90]
	Proteasome mediated	[64]
BCL3	Protein Stabilization	[91]
PKA	CtBP dimerization	[92]
AMPK1	Phosphorylation dependent inactivation	[93]
XIAP	Polyubiquitination degradation	[94]
Stub1/CHIP	CtBP2 polyubiquitination degradation	[95]
AKT1	Phosphorylation induced decreased dimerization	[96]
JNK1	Phosphorylation dependent	[66]
	Proteasomal degradation	
PAK1	Phosphorylation dependent	[97]
	Translocation to the cytoplasm	
CBX4/UBC9	Sumoylation-dependent nuclear retention	[98–101]
p300	Acetylation increased nuclear retention	[102]
